# Supplementary material for: Distinct cortical systems reinstate the content and context of episodic memories
Source: Nat Commun. 2021 Jul 21;12:4444. doi: 10.1038/s41467-021-24393-1 (PMC8295370; doi:10.1038/s41467-021-24393-1)
Supplement: Supplementary file 1 — Supplementary Information [file 41467_2021_24393_MOESM1_ESM.pdf]

Supplementary Information: Distinct cortical systems reinstate the content and context of episodic memories

James E. Kragel, Youssef Ezzyat, Bradley C. Lega, Michael R. Sperling, Gregory A. Worrell, Robert E. Gross, Barbara C. Jobst, Sameer A. Sheth, Kareem A. Zaghloul, Joel M. Stein, Michael J. Kahana

Supplementary Figures

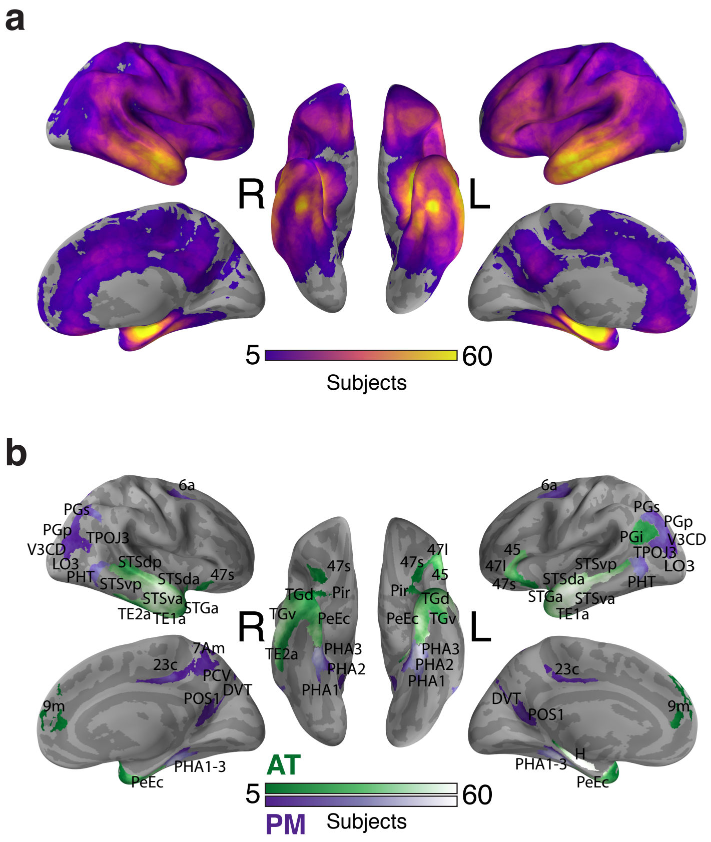

Supplementary Figure 1: **Electrode coverage.** **a**, Surface renders depict the number of subjects with electrode contacts within 10 mm of a vertex, after transformation into MNI space. **b**, The plot depicts the coverage as in **a**, highlighting parcels assigned to either the anterior temporal (AT) or posterior medial (PM) network. Parcel naming convention follows Glasser et al., 2016[30].

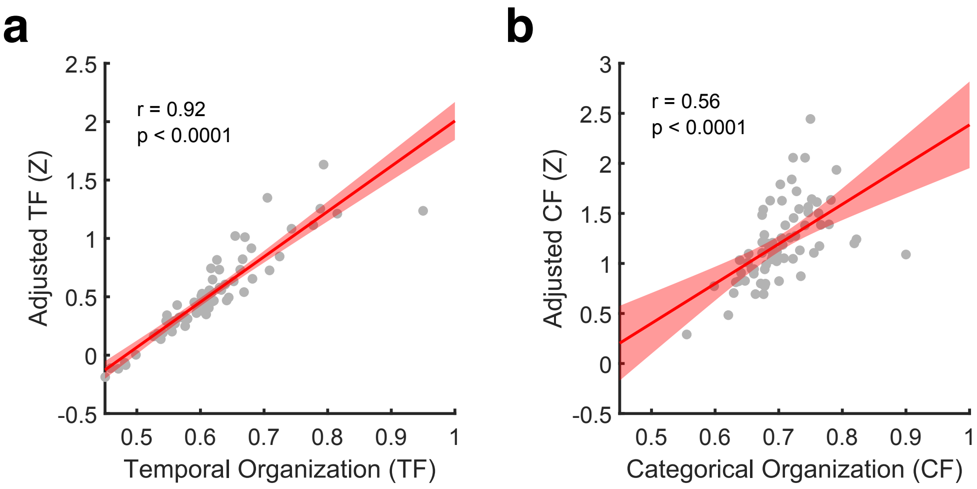

Supplementary Figure 2: **Measures of temporal and categorical organization are robust to list structure.** **a**, The scatter plot depicts the observed temporal factor (TF) score for each patient and the standardized TF score (in units of one standard deviation) after adjusting for the amount of temporal organization expected by chance from the category structure of each list. The red line indicates least squares linear fit across the group. **b**, The scatter depicts observed and adjusted category factor (CF) scores, accounting for the category structure of presented lists. Each point indicates a single subject. Shaded regions denote mean ± SEM ($n = 69$ subjects). Statistical inference was performed with two-sided Student's t tests (**a** and **b**).

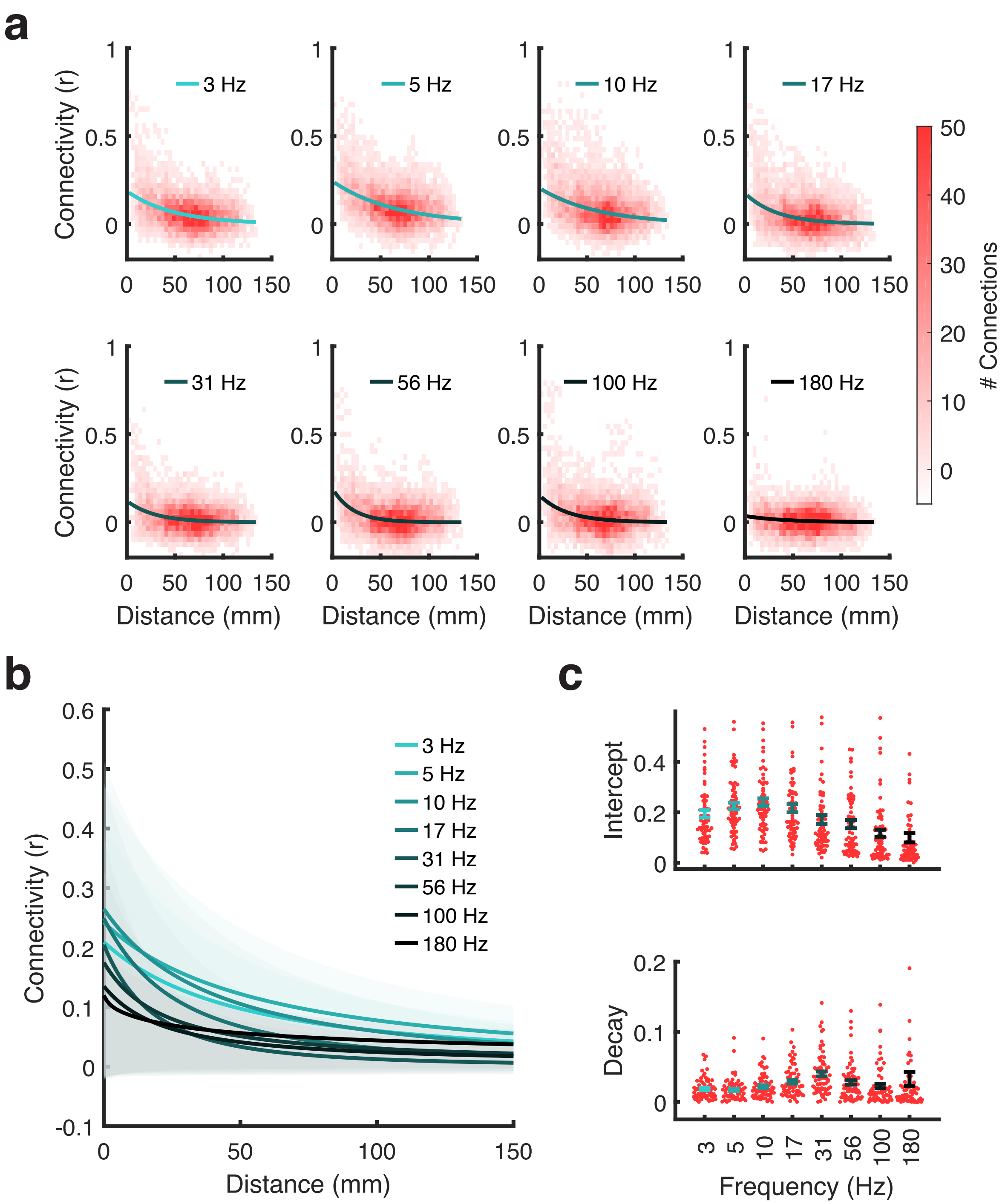

Supplementary Figure 3: **Connectivity between recording sites decreases with distance.** **a**, Plots depict intrinsic connectivity as a function of inter-electrode difference for an individual subject. Best fit exponentials are overlaid on each plot. **b**, Group-averaged ($n = 69$ subjects) predictions on the effect of inter-electrode distance on intrinsic connectivity. Shaded regions denote mean $\pm$ SEM. **c**, Lines depict group-averaged model parameters for describing the relation between distance and connectivity. Each point indicates a single subject (total $n = 69$ subjects). Error bars denote SEM.

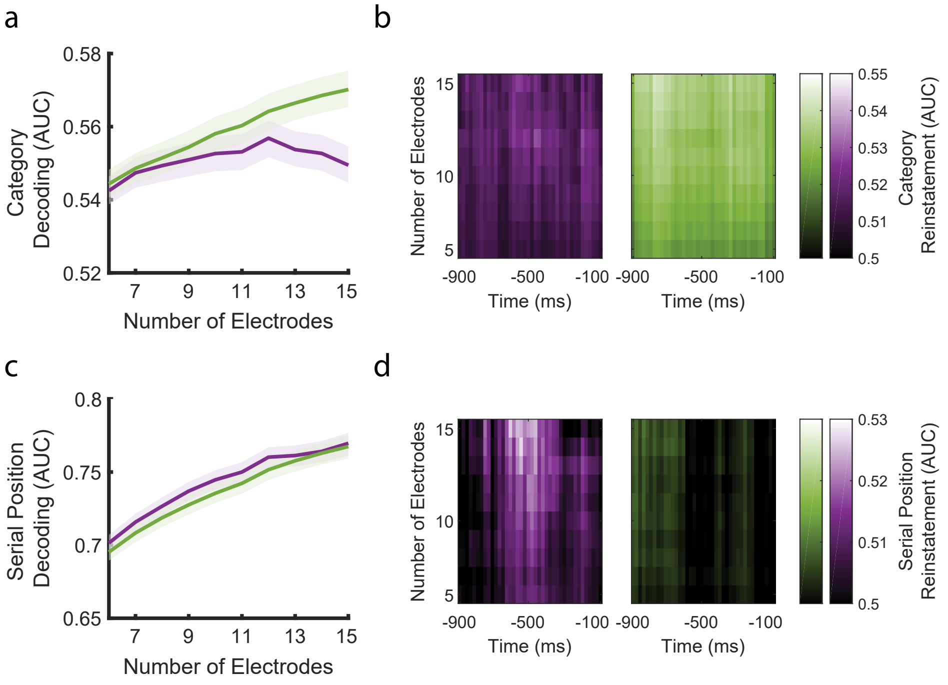

Supplementary Figure 4: **Decoding accuracy increases with electrode coverage.** **a**, Line plots depict the average category decoding as a function of the number of electrodes sampled from the PM (purple) and AT (green) networks. Shaded regions denote mean $\pm$ SEM ($n = 69$ subjects). **b**, Each map depicts category reinstatement in the moments leading up to item recall, within each network. Changes in serial position decoding during encoding (**c**) and associated reinstatement effects (**d**) follow the same plotting convention. Shaded regions denote mean $\pm$ SEM ($n = 69$ subjects).

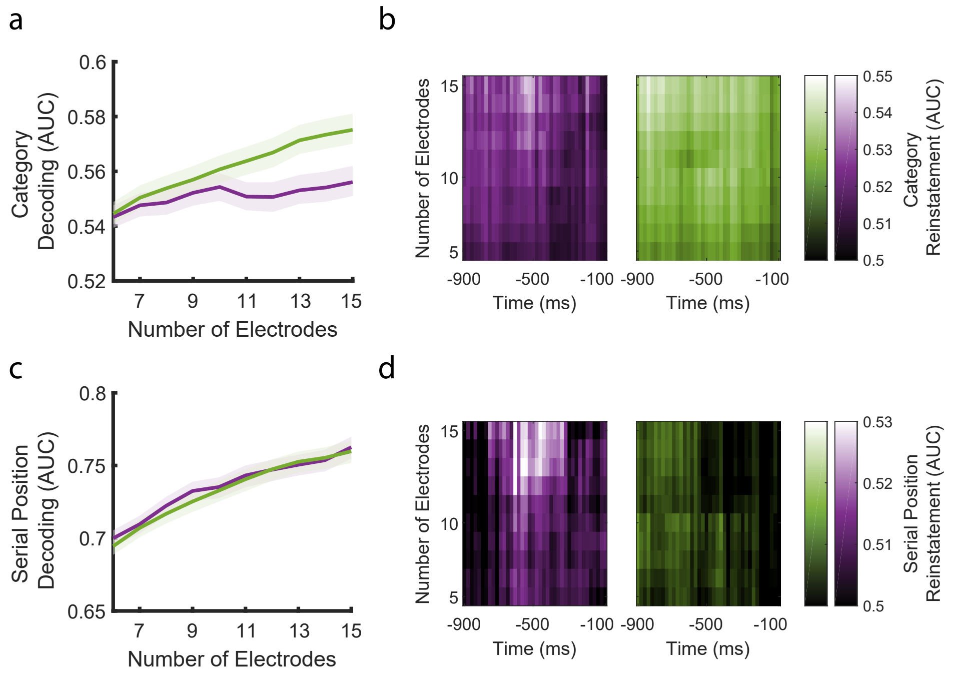

Supplementary Figure 5: **Reinstatement effects are not influenced by epileptogenic brain regions.** Excluding electrodes from the seizure onset zone does not impact category decoding during encoding (**a**) or recall (**b**). Shaded regions denote mean ± SEM ($n = 69$ subjects). The same pattern of results holds for decoding serial position of encoded items (**c**) and reinstatement of related activity (**d**). Shaded regions denote mean ± SEM ($n = 69$ subjects).
