# Supplementary material for: Distinct cortical systems reinstate the content and context of episodic memories
Source: Nat Commun. 2021 Jul 21;12:4444. doi: 10.1038/s41467-021-24393-1 (PMC8295370; doi:10.1038/s41467-021-24393-1)
Supplement: Supplementary file 3 — Reporting Summary [file 41467_2021_24393_MOESM3_ESM.pdf]

Corresponding author(s): Michael Kahana
James Kragel

Last updated by author(s): Feb 22, 2021

Reporting Summary

Nature Research wishes to improve the reproducibility of the work that we publish. This form provides structure for consistency and transparency in reporting. For further information on Nature Research policies, see our Editorial Policies and the Editorial Policy Checklist.

Statistics

For all statistical analyses, confirm that the following items are present in the figure legend, table legend, main text, or Methods section.

n/a	Confirmed	
☐	☒	The exact sample size (*n*) for each experimental group/condition, given as a discrete number and unit of measurement
☐	☒	A statement on whether measurements were taken from distinct samples or whether the same sample was measured repeatedly
☐	☒	The statistical test(s) used AND whether they are one- or two-sided<br>*Only common tests should be described solely by name; describe more complex techniques in the Methods section.*
☐	☒	A description of all covariates tested
☐	☒	A description of any assumptions or corrections, such as tests of normality and adjustment for multiple comparisons
☐	☒	A full description of the statistical parameters including central tendency (e.g. means) or other basic estimates (e.g. regression coefficient) AND variation (e.g. standard deviation) or associated estimates of uncertainty (e.g. confidence intervals)
☐	☒	For null hypothesis testing, the test statistic (e.g. *F*, *t*, *r*) with confidence intervals, effect sizes, degrees of freedom and *P* value noted<br>*Give P values as exact values whenever suitable.*
☒	☐	For Bayesian analysis, information on the choice of priors and Markov chain Monte Carlo settings
☐	☒	For hierarchical and complex designs, identification of the appropriate level for tests and full reporting of outcomes
☐	☒	Estimates of effect sizes (e.g. Cohen's *d*, Pearson's *r*), indicating how they were calculated

Our web collection on statistics for biologists contains articles on many of the points above.

Software and code

Policy information about availability of computer code

Data collection	Custom python (3.3, with the PyEPL package) software was used for experiment control.
Data analysis	Custom Python (3.3) code was used for preprocessing, including both electrophysiological data analysis (https://github.com/pennmem/ptsa_new) and electrode localization (https://github.com/pennmem/neurorad_pipeline). Electrode localization also relied on Freesurfer (v1.379.2.73) for cortical surface estimation.<br><br>Further data analysis was conducted using MATLAB r2017b and custom code (memory.psych.upenn.edu/files/pubs/KragEtal21.code.tgz).

For manuscripts utilizing custom algorithms or software that are central to the research but not yet described in published literature, software must be made available to editors and reviewers. We strongly encourage code deposition in a community repository (e.g. GitHub). See the Nature Research guidelines for submitting code & software for further information.

Data

Policy information about availability of data

All manuscripts must include a data availability statement. This statement should provide the following information, where applicable:
- Accession codes, unique identifiers, or web links for publicly available datasets
- A list of figures that have associated raw data
- A description of any restrictions on data availability

De-identified data is available at http://memory.psych.upenn.edu/Electrophysiological_Data. Source data are provided with this paper.

Field-specific reporting

Please select the one below that is the best fit for your research. If you are not sure, read the appropriate sections before making your selection.

☒ Life sciences ☐ Behavioural & social sciences ☐ Ecological, evolutionary & environmental sciences

For a reference copy of the document with all sections, see nature.com/documents/nr-reporting-summary-flat.pdf

Life sciences study design

All studies must disclose on these points even when the disclosure is negative.

Sample size	No sample-size calculation was performed to determine the number of subjects. Data were collected as part of a separate large-scale study to modulate memory function with direct brain stimulation. All participants with electrode coverage within brain networks of interest were included for analysis. The resulting sample size was an order of magnitude greater than many similar studies, and we used all available data at the time of analysis.
Data exclusions	The main results reported in the manuscript did not include any data exclusions. As a control analysis, we excluded any electrode contacts within the seizure onset zone or atypical tissue to rule out the possibility that our results were influenced by epileptogenic activity.
Replication	We did not replicate the present findings in an independent cohort, given the rarity of intracranial recordings in human patients.
Randomization	Randomization was not relevant to our study as we used a single group, within-subject design.
Blinding	Blinding was not relevant to our study as we used a single group, within-subject design.

Reporting for specific materials, systems and methods

We require information from authors about some types of materials, experimental systems and methods used in many studies. Here, indicate whether each material, system or method listed is relevant to your study. If you are not sure if a list item applies to your research, read the appropriate section before selecting a response.

Materials & experimental systems

n/a	Involved in the study	
☒	☐	Antibodies
☒	☐	Eukaryotic cell lines
☒	☐	Palaeontology and archaeology
☒	☐	Animals and other organisms
☐	☒	Human research participants
☒	☐	Clinical data
☒	☐	Dual use research of concern

Methods

n/a	Involved in the study	
☒	☐	ChIP-seq
☒	☐	Flow cytometry
☒	☐	MRI-based neuroimaging

Human research participants

Policy information about studies involving human research participants

Population characteristics	69 patients (40 male, with an average age of 39 years [SD 12 years]) with medication-resistant epilepsy participated in the study. On average, the duration of epilepsy at the time of surgery was 19 years (SD 13 years). Seizure onset zones were commonly localized to mesial temporal (N=25), temporal (N=22), prefrontal (13), parietal (N=28), and occipital (N=7) cortices. An additional two patients had seizure onset zones within the hippocampus. Seizure onset zones were left-lateralized (N=31), right-lateralized (N=23), or bilateral (N=11).
Recruitment	Eligible patients were recruited at each participating research site by clinical teams and neurosurgeons. Although no strict neuropsychiatric cutoffs were used, patients were recruited based on their cognitive function and ability to perform memory tasks. As a result, our sample is likely to reflect patients with limited impairments in memory, which is commonplace in epilepsy.
Ethics oversight	Our research protocol was approved by the Institutional Review Board at each participating university prior to data collection, including Dartmouth-Hitchcock Medical Center (Hanover, NH), Emory University Hospital (Atlanta, Georgia), Hospital of the University of Pennsylvania (Philadelphia, PA), Mayo Clinic (Rochester, MN), Thomas Jefferson University Hospital (Philadelphia, PA), Columbia University Medial Center (New York, NY), and University of Texas Southwestern Medical Center (Dallas, TX).

Note that full information on the approval of the study protocol must also be provided in the manuscript.
